# Mechanism and Function of Mixed-Mode Oscillations in Vibrissa Motoneurons

**DOI:** 10.1371/journal.pone.0109205

**Published:** 2014-10-02

**Authors:** David Golomb

**Affiliations:** Departments of Physiology and Cell Biology, Physics and Zlotowski Center for Neuroscience, Faculty of Health Sciences, Ben Gurion University, Be'er-Sheva, Israel; McGill University, Canada

## Abstract

Vibrissa motoneurons in the facial nucleus innervate the intrinsic and extrinsic muscles that move the whiskers. Their intrinsic properties affect the way they process fast synaptic input from the vIRT and Bötzinger nuclei together with serotonergic neuromodulation. In response to constant current (*I*_app_) injection, vibrissa motoneurons may respond with mixed mode oscillations (MMOs), in which sub-threshold oscillations (STOs) are intermittently mixed with spikes. This study investigates the mechanisms involved in generating MMOs in vibrissa motoneurons and their function in motor control. It presents a conductance-based model that includes the M-type K^+^ conductance, *g*_M_, the persistent Na^+^ conductance, *g*_NaP_, and the cationic h conductance, *g*_h_. For *g*_h_ = 0 and moderate values of *g*_M_ and *g*_NaP_, the model neuron generates STOs, but not MMOs, in response to *I*_app_ injection. STOs transform abruptly to tonic spiking as the current increases. In addition to STOs, MMOs are generated for *g*_h_>0 for larger values of *I*_app_; the *I*_app_ range in which MMOs appear increases linearly with *g*_h_. In the MMOs regime, the firing rate increases with *I*_app_ like a Devil's staircase. Stochastic noise disrupts the temporal structure of the MMOs, but for a moderate noise level, the coefficient of variation (CV) is much less than one and varies non-monotonically with *I*_app_. Furthermore, the estimated time period between voltage peaks, based on Bernoulli process statistics, is much higher in the MMOs regime than in the tonic regime. These two phenomena do not appear when moderate noise generates MMOs without an intrinsic MMO mechanism. Therefore, and since STOs do not appear in spinal motoneurons, the analysis can be used to differentiate different MMOs mechanisms. MMO firing activity in vibrissa motoneurons suggests a scenario in which moderate periodic inputs from the vIRT and Bötzinger nuclei control whisking frequency, whereas serotonergic neuromodulation controls whisking amplitude.

## Introduction

Motoneurons in the facial nucleus [1] innervate the intrinsic and extrinsic muscles that move the whiskers of rodents [2]–[6]. The frequency range of exploratory whisking in rats is 5–15 Hz [4], and the firing rate of vibrissa motoneurons can be as low as a few Hz [7], [8]. These motoneurons are not mutually coupled, and are innervated by many brainstem areas [9]. Motoneurons that project to intrinsic and extrinsic muscles receive rhythmic synaptic input from the vIRT and Bötzinger nuclei respectively [10], [11], and both types of motoneurons are under the modulation of serotonin and other neuromodulators [8], [12]. It is still unclear how those inputs are integrated, or the ways in which integration is affected by the intrinsic properties of vibrissa motoneurons.

Intrinsic neuronal properties are often examined in *in vitro* experiments. Like other neurons, vibrissa motoneurons fire tonically under the application of applied current *I*_app_ if *I*_app_ is above a critical value. Below this value, they may fire in mixed mode oscillations (MMOs) [13], an alternation of subthreshold oscillations (STOs) with spiking behavior (Figure 3A in [12] and Figure 5A in [14]). At first glance, the inter-spike interval during these MMO states appears irregular. From an ionic current perspective, the firing of vibrissa motoneurons depends strongly on the level of the persistent Na^+^ conductance g_NaP_ [8], and enhancing g_NaP_ by a serotonergic agonist generates rhythmic firing in vibrissa motoneurons. Spiking in vibrissa motoneurons is followed by pronounced afterhyperpolarization, and the low firing rate is explained by AHP conductances that are significantly slower than AHP conductances in spinal motoneurons [8], [15]. These neurons also exhibit an h-conductance and response in that they “sag” to hyperpolarizing current steps [16].

In addition to rat vibrissa motoneurons, MMOs have been found in mouse [17] and rat [18] spinal motoneurons. In spinal motoneurons, the firing frequency in the tonic regime (“primary range”), as well as the frequency of the STOs between spikes in the MMO regime (“sub-primary range) were reported to be about 40–100 Hz [19]. Fast STOs in spinal motoneurons can be generated from the interplay between the fast, transient sodium current and two potassium currents: the delayed-rectifier current and an afterhyperpolarization (AHP) current. The time scale of the first two currents and the third current were reported to be 1 and 10 ms, respectively. MMOs were found to emerge from the Shilnikov bifurcation of the resting state [19].

The current study deals with four unanswered questions: Which mechanisms can lead to MMOs generation in vibrissa motoneurons? How can they be distinguished based on the properties of their firing patterns? Is the mechanism in vibrissa motoneurons different from that of spinal motoneurons [19]? What are the functional advantages for motoneurons to be in the MMO mode? This is done by analyzing a conductance-based model in vibrissa motoneurons that can exhibit MMO activity [15].

## Results

### Model of a vibrissa motoneuron

The single compartment, conductance-based model for a vibrissa motoneuron was described in [15]. In brief, the current balance equation is 
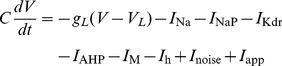
(1)where V is the membrane potential of the neuron, C = 1 µF/cm^2^ is the membrane capacitance of the neuron, and the parameters of the leak current are *g*_L_ = 0.12 mS/cm^2^ and *V*_L_ = −70 mV (see “Ionic currents of the model” in Methods). The applied current injected into the neuron is denoted by *I*_app_, which is considered to be constant in time unless otherwise stated. The ionic currents consist of the transient Na^+^ current, *I*_Na_; the persistent Na^+^ current, *I*_NaP_; the delayed rectifier K^+^ current, *I*_Kdr_; the slow AHP, Ca^2+^-dependent K^+^ current, *I*_AHP_; the slow voltage dependent, K^+^ current, *I*_M_; and the hyperpolarization-activated h-current *I*_h_. The noise current is 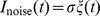 [20], where *ξ*(*t*) is a Gaussian white noise: 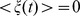 and 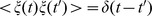; <…> means average over trials and *δ* is the Dirac function (see [21]). The units of *σ* are µA×ms^1/2^/cm^2^. The value of *σ* = 0 is used unless stated otherwise.

### STOs in noiseless neurons with *g*_h_ = 0

**Figure 1:**
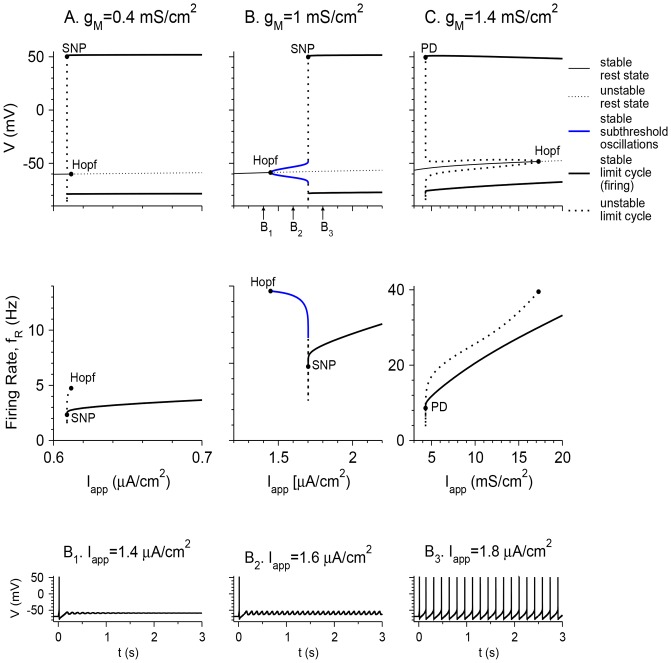
Bifurcation diagrams of the vibrissa motoneuron model with *g*_h_ = 0. The values of the membrane potential *V* (top panels) and the firing rate *f*_R_ (medium panels) are plotted as functions of *I*_app_ for fixed points (thin lines) and limit cycles (thick lines) for *g*_M_ = 0.4 mS/cm^2^ (A), *g*_M_ = 1 mS/cm^2^ (B) and *g*_M_ = 1.4 mS/cm^2^ (C). For limit cycles, minimal and maximal voltages during the cycle are plotted. Solid lines denote stable solutions, and dotted lines denote unstable solutions. Stable sub-threshold oscillations are shown in blue, whereas stable tonic firing states are shown in solid thick black lines. Solid circles denote bifurcations from the following types: Hopf (HB), saddle-node of periodics (SNP) and period doubling (PD). Panels B_1_-B_3_ at the bottom present the voltage time traces for *g*_M_ = 1 mS/cm^2^ and *I*_app_ = 1.4, 1.6 and 1.8 µA/cm^2^ respectively. These *I*_app_ values are denoted by the arrows below the abscissa in panel B (top).

The first step is to investigate the neuronal dynamics with no h-conductance. This case is studied for two reasons. First, it is simpler than the case with *g*_h_>0. Second, the dynamical regimes defined for *g*_h_ = 0 provide the basis over which more complicated dynamics for *g*_h_>0 are identified. The relationship between the firing pattern and *I*_app_ in the model depends on the M-type K^+^ conductance, *g*_M_. For small *g*_M_, there is a transition from a rest state to a firing state as *I*_app_ increases. For example, the bifurcation diagram for *g*_M_ = 0.4 mS/cm^2^ (Figure 1A) shows that the rest state is destabilized via a subcritical Hopf bifurcation, and a firing state with a frequency of a few Hz emerges. There is a narrow bistable regime [22]. At intermediate *g*_M_ values, such as *g*_M_ = 1 mS/cm^2^ (Figure 1B), the rest state is destabilized via a supercritical Hopf bifurcation, and a state of STOs emerges. This state switches abruptly to a firing state at a large *I*_app_ value. This abrupt switch corresponds to the canard scenario [23]. Examples of convergence to these three states – rest, STO and firing – in response to step currents are shown in Figures 1B_1-3_. When *g*_M_ further increases, the resting state is destabilized again by a subcritical Hopf bifurcation, this time with an extended bistable regime (Figure 1C), and there are no STOs.

**Figure 2:**
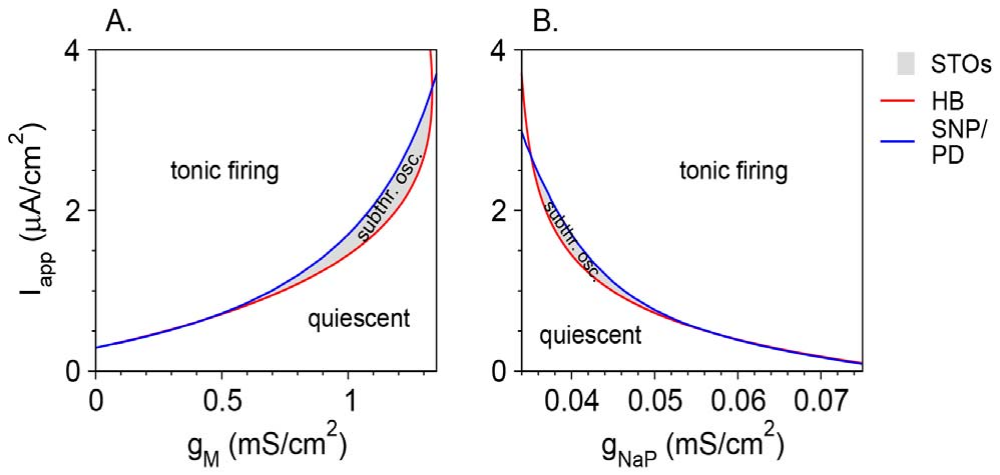
Phase diagrams of the vibrissa motoneuron model with *g*_h_ = 0. The dynamical states of the model neuron are plotted in the *g*_M_-*I*_app_ plane (A) and in the *g*_M_-*I*_app_ plane (B). A regime of STOs (light grey) is obtained between the regimes of quiescence and tonic firing. Red lines denote the Hopf bifurcation (HB), and blue lines denote the saddle-node of periodics (SNP) or period doubling (PD) bifurcations.

The phase diagram in the *I*_app_–*g*_M_ plane shows that STOs are obtained in a restricted *I*_app_ regime, between a regime of quiescence and a regime of tonic firing. For STOs to occur, *g*_M_ should be neither too small nor too large (0.42 mS/cm^2^ <*g*_M_<1.33 mS/cm^2^; Figure 2A). The *I*_app_ value for which STOs emerge increases with *g*_M_. The *I*_app_ range in which STOs occur increases and then decreases with *g*_M_. For STOs to occur, the persistent sodium conductance *g*_NaP_ should also be within intermediate values (0.035 mS/cm^2^ <*g*_NaP_ <0.057 mS/cm^2^; *g*_M_ = 1 mS/cm^2^; Figure 2B). The *I*_app_ value for which STOs emerge decreases with *g*_NaP_. The *I*_app_ range in which STOs occur increases and then decreases with *g*_NaP_. No MMOs were observed in the noiseless model without *g*_h._

### MMOs in noiseless neurons with *g*_h_

**Figure 3:**
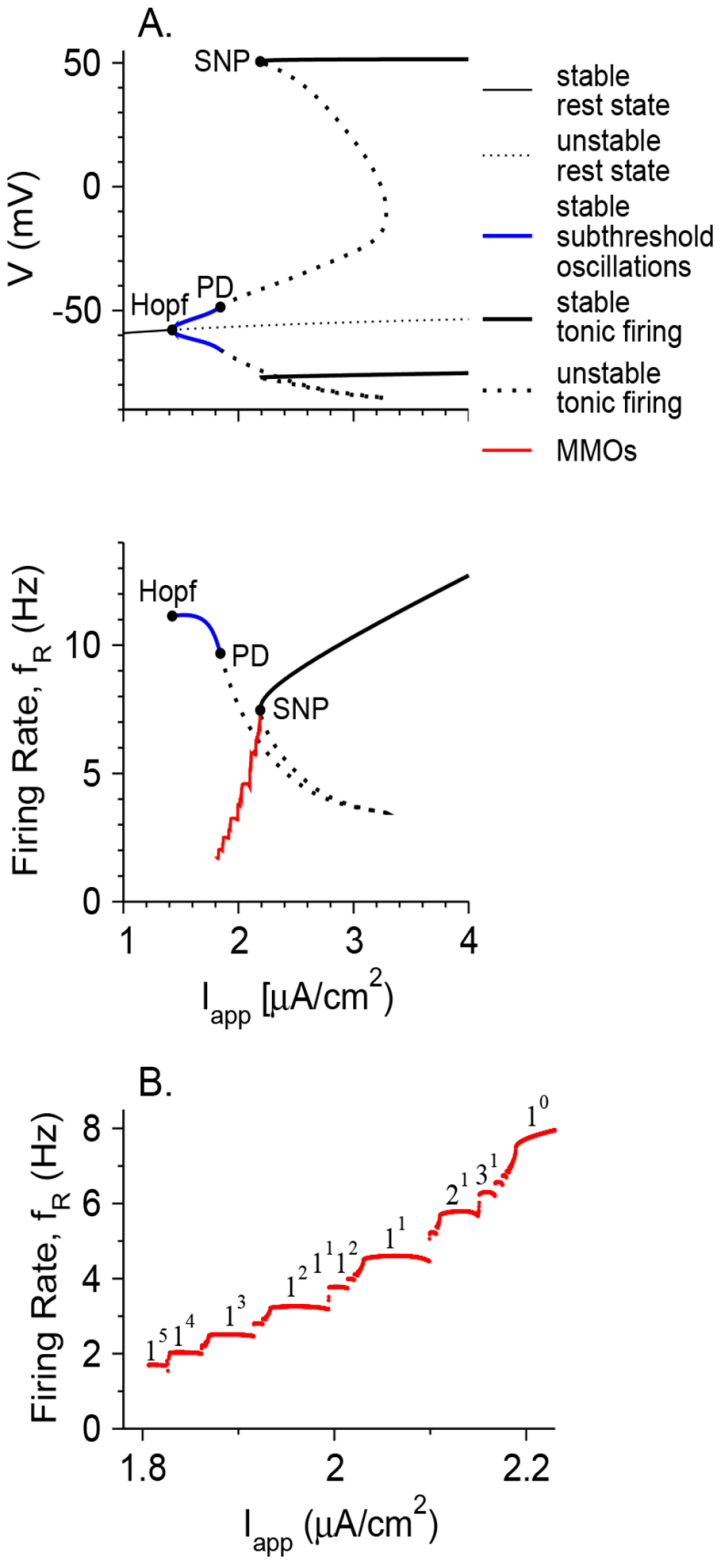
Bifurcation diagrams of the vibrissa motoneuron model with *g*_h_ = 0.3 mS/cm^2^. (A) The values of the membrane potential *V* (top panel) and the firing rate *f*_R_ (bottom panel) are plotted as functions of *I*_app_ for fixed points (thin lines) and limit cycles (thick lines) for *g*_M_ = 1 mS/cm^2^. For limit cycles, minimal and maximal voltages during the cycle are plotted. Solid lines denote stable solutions, and dotted lines denote unstable solutions. Stable sub-threshold oscillations are shown in blue, whereas stable tonic firing states are shown in solid thick black lines. Solid circles in the top panels denote bifurcations from the following types: Hopf (HB), saddle-node of periodics (SNP) and period doubling (PD). The firing rate in the MMOs state is plotted in red in the bottom panel. (B) The firing rate *f*_R_ in the MMOs state is plotted as a function of *I*_app_ at a larger scale. The types of mixed mode states (see text, Figure 4 and [23]) are indicated above the curve.

**Figure 4:**
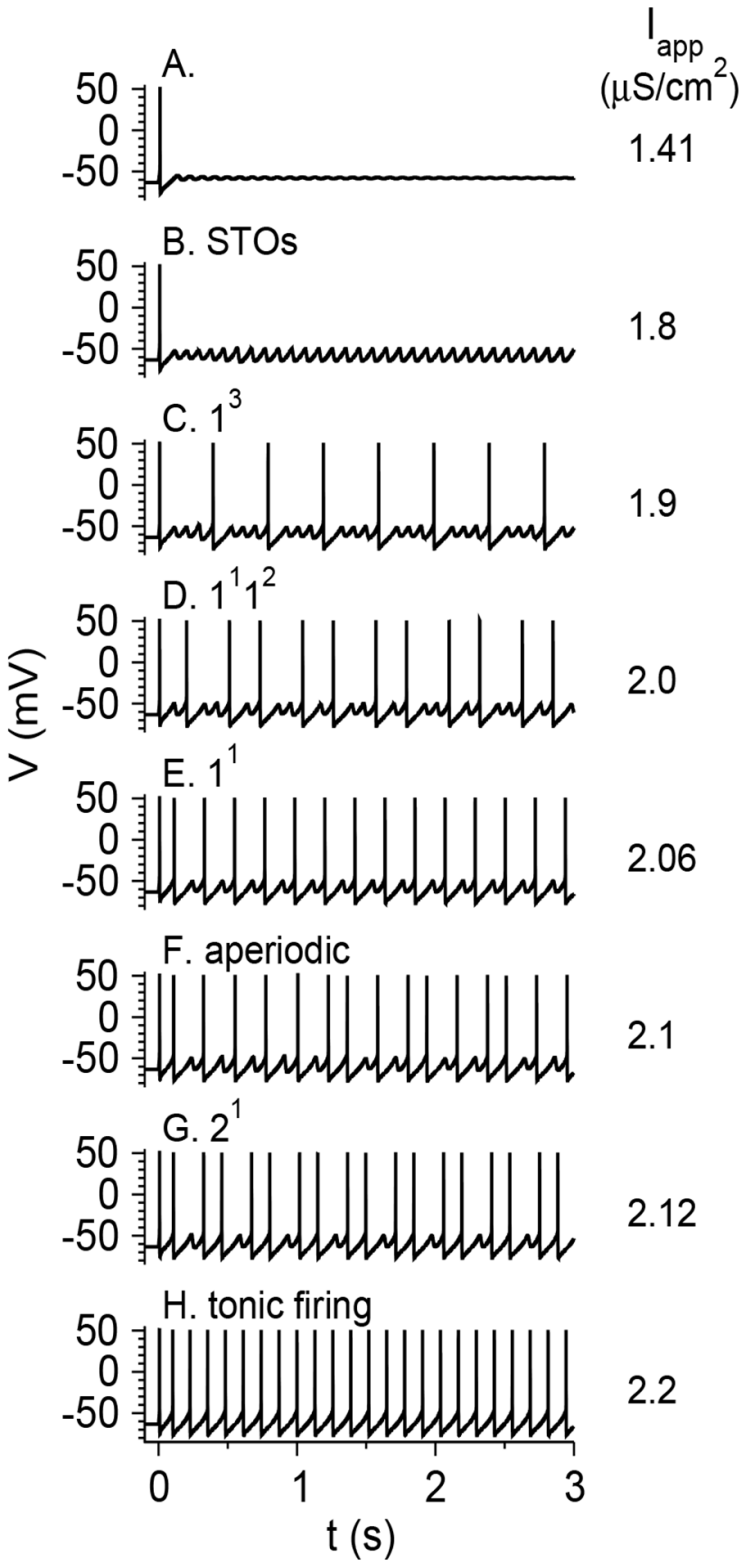
Voltage time traces of the model neuron in response to step current injection at *t* = 0. Parameters are as in Figure 3 (*g*_h_ = 0.3 mS/cm^2^). The values of *I*_app_ are written in units of µA/cm^2^. (A) *I*_app_ = 1.41, the membrane potential of the neuro goes to rest. (B) *I*_app_ = 1.8, the neuron exhibits sub-threshold oscillations. (C) *I*_app_ = 1.9, the neuron fires in an MMOs mode, with 3 sub-threshold oscillations between each pair of consecutive spikes. (D) *I*_app_ = 2.0, the neuron fires in an MMOs mode. The number of STOs between pairs of consecutive spikes switches alternately between 1 and 2. (E) *I*_app_ = 2.06, the neuron fires in an MMOs mode with one STO between two consecutive spikes. (F) *I*_app_ = 2.1, the neuron fires aperiodically. (G) *I*_app_ = 2.12, The neuron fires two spikes, shows one STO, and then the cycle starts again. (H) *I*_app_ = 2.2, the neuron fires tonically. The dynamical states are indicated above each panel.

When the h conductance *g*_h_ is larger than zero, bifurcation diagrams of the model neurons (Figure 3A) resemble those for the case where *g*_h_ = 0 (Figure 1B). For the reference parameter set (*g*_M_ = 1 mS/cm^2^), the system switches from a rest state (Figure 4A) to STOs (Figure 4B) as *I*_app_ increases. These STOs are destabilized via a period doubling (PD) bifurcation at *I*_app_ = *I*_PD_. At *I*_app_ = *I*_SNP_ (*I*_SNP_>*I*_PD_) there is a saddle-node of periodics (SNP) bifurcation. The neuron fires tonically for *I*_app_>*I*_SNP_ (Figure 4H). There is, however, one major difference between the model with and without *g*_h_. For *g*_h_>0, there is a range of *I*_app_ between *I*_PD_ and *I*_SNP_ where no simple attractor (rest state or tonic firing) exists. Instead, the model neuron exhibits MMOs characterized by subthreshold oscillations between spikes (Figures 3, 4C–G).

The number of STOs between two consecutive spikes decreases with *I*_app_, whereas the STO frequency depends only weakly on *I*_app_ (Figure 4). As a result, the average firing rate during the MMO state increases with *I*_app_ (Figure 3B). In most cases (e.g., Figure 4C–E,G), but not all (e.g., Figure 4F) the MMO state is periodic. The labeling method of [23] is thus modified to define the state during periodic MMOs by indicating the number of spikes (as regular numbers) and STOs (as superscripts) in consecutive episodes within one time period. The states shown in Figure 4B–H are therefore: B. 0^1^ is (an STO state). C. 1^3^. D. 1^1^1^2^. E. 1^1^. F. Aperiodic state. G. 2^1^. H. 1^0^ (tonic firing). The 1^1^ state occupies the largest *I*_app_ range (Figure 3B). For smaller values of *I*_app_, the firing pattern resembles 1^n^ for most of the *I*_app_ interval, where *n* is an integer. For the reference parameter set, the maximal *n* is 5. There are narrower ranges where the firing patterns are more complicated, such as 1^1^1^2^. Aperiodic, probably chaotic patterns are also found. For *I*_app_ values larger than that of the 1^1^ range, the neuron fires mostly in *n* ^1^ patterns, and *n* increases with *I*_app_. For large enough *I*_app_ the firing patterns switch to 1^0^ and the neuron fires tonically without STOs between spikes. The graph of the firing rate *f*_R_ as a function of *I*_app_ (Figure 3B) has a Devil's staircase shape [24].

**Figure 5:**
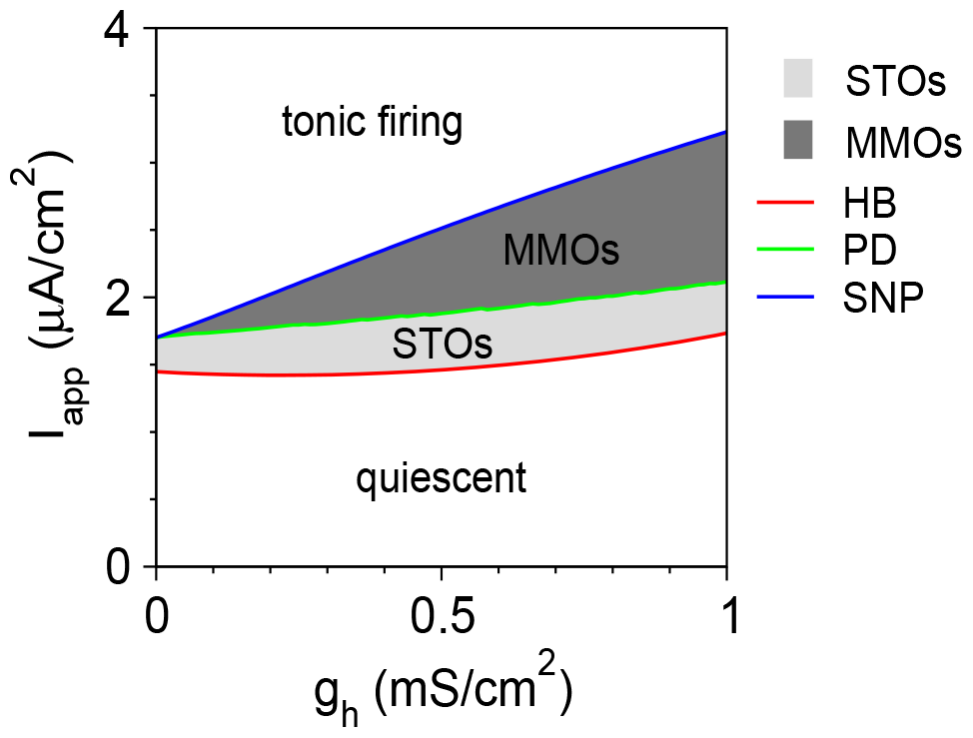
Phase diagram of the vibrissa motoneuron model in the *g*_h_-*I*_app_ plane. Regimes of STOs (light grey) and MMOs (dark grey) are obtained between the regimes of quiescence and tonic firing. The red line denotes Hopf bifurcation (HB), the green line denotes period doubling (PD) bifurcation, and the blue line denotes saddle-node of periodics (SNP) bifurcation.

The phase diagram in the *I*_app_–*g*_h_ plane (Figure 5) shows that MMOs are obtained between an STO regime and a tonic firing regime. The *I*_app_ range in which MMOs are obtained increases approximately linearly with *g*_h_; it approaches 0 as *g*_h_ →0. The *I*_app_ range in which the neuron exhibits STOs varies only weakly with *g*_h_.

### Noise-induced MMOs

**Figure 6:**
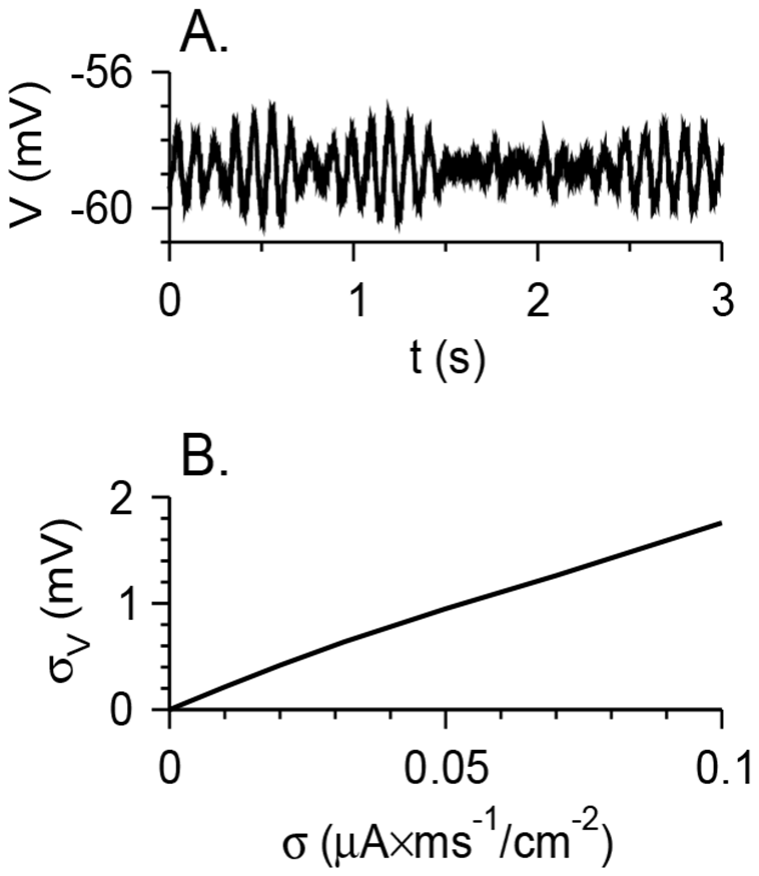
Voltage fluctuations generated by stochastic noise. (A) Voltage time traces of the model neurons with *g*_M_ = 1 mS/cm^2^, *g*_NaP_ = 0.04 mS/cm^2^, *g*_h_ = 0, *I*_app_ = 1.4 µA/cm^2^, σ = 0.032 µA×ms^1/2^/cm^2^. For σ = 0, the model neurons are at rest for this parameter set. With noise, the membrane potential fluctuates. (B) The standard deviation of the voltage σ_V_ as a function of the noise level σ. This figure demonstrates how the noise strength affects the magnitude of voltage fluctuations without any intrinsic STOs mechanism.

Individual ionic channels are discrete elements whose properties can be given only probabilistically [25]. Spontaneous synaptic release is also probabilistic [26]. In neuronal modeling, this stochastic dynamics are often modeled by adding white stochastic, Gaussian noise to the underlying deterministic dynamics [20]. Here, a second mechanism for MMOs generation is considered, in which stochastic noise leads to MMOs firing patterns when the noiseless neuron exhibits STOs or even quiescence or tonic firing. To analyze noise effects, the properties of the motoneuron model with a nonzero noise amplitude σ were studied. To show the impact of parameter σ, the model was simulated in a regime where it is at rest for σ = 0 (no spikes, STOs or MMOs). Here, weak or moderate noise generates voltage fluctuation but not spikes (Figure 6A). The standard deviation of the fluctuations σ_V_ is defined as
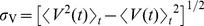
(2)where 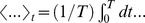 means time average over a large time *T*. The standard deviation σ_V_ increases linearly with σ for small σ (Figure 6B), and spikes are observed for large enough σ. Spikes are not observed for σ values below 0.1 µA×ms^1/2^/cm^2^, that yield voltage fluctuations below 2 mV.

**Figure 7:**
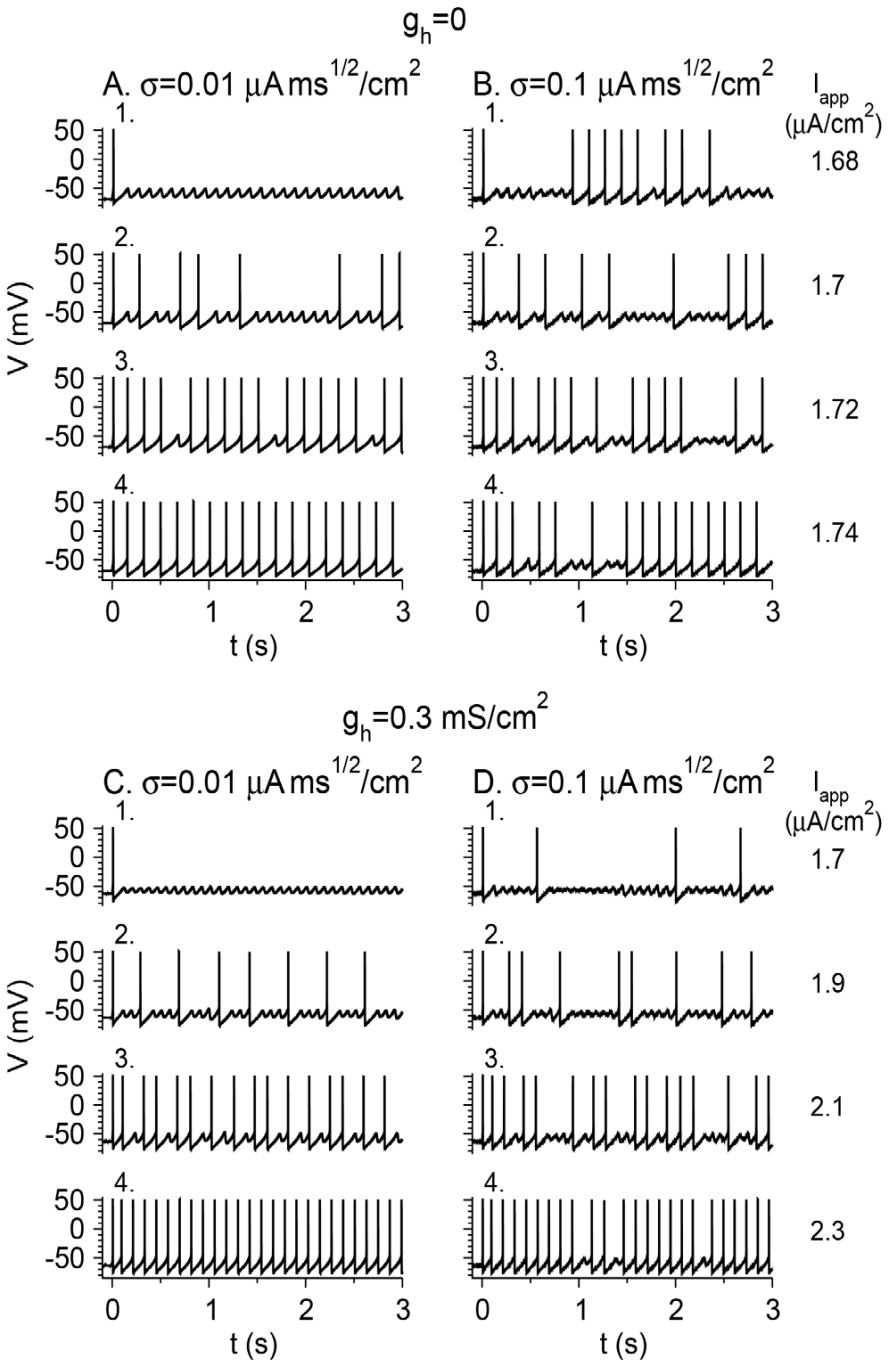
Voltage time traces of the model neuron in response to step current injection at *t* = 0. The values of *I*_app_ are indicated to the right of the traces. (A) *g*_h_ = 0, σ = 0.01 µA×ms^1/2^/cm^2^. The noiseless neuron does not exhibit MMOs, but this level of noise generates MMOs near the transition between quiescence and tonic firing. (B) *g*_h_ = 0, σ = 0.1 µA×ms^1/2^/cm^2^. For this larger noise level, MMOs are generated in a more widespread *I*_app_ regime. (C) *g*_h_ = 0.3 mS/cm^2^, σ = 0.01 µA×ms^1/2^/cm^2^. The noiseless neuron generates MMOs. This level of noise increases the *I*_app_ regime in which MMOs are obtained only slightly. The MMOs are less ordered, and the number of STOs between spikes varies from one inter-spike interval to another. (D) *g*_h_ = 0.3 mS/cm^2^, σ = 0.1 µA×ms^1/2^/cm^2^. MMOs appear in *I*_app_ regimes in which the noiseless neuron is quiescent or fires tonically, and the firing patterns look less ordered.

Noise may generate MMOs when they do not exist for the noiseless neuron, or increase their range of appearance when they do. First, I study a case in which the noiseless neuron does not exhibit MMOs: *g*_h_ = 0, *g*_M_ = 1 mS/cm^2^. It exhibits STOs for *I*_app_ between *I*_HB_ = 1.45 µA/cm^2^ and *I*_PD_ = 1.7 µA/cm^2^ (Fig. 1B). It spikes tonically for *I*_app_>*I*_PD_. A (relatively small) noise level of σ = 0.01 µA×ms^1/2^/cm^2^ transfers STOs or tonic spiking activity to MMOs in the *I*_app_ in a restricted range around *I*_PD_ (Figure 7A_2,3_), but not far from that value (Figure 7A_1,4_). Increasing σ expands the range of noise-generated MMOs (σ = 0.1 µA×ms^1/2^/cm^2^; Figure 7B). Second, I study a case in which the noiseless neuron can exhibit MMOs: *g*_h_ = 0.3 mS/cm^2^, *g*_M_ = 1 mS/cm^2^ (Figure 5). Small levels of noise such as σ = 0.01 µA×ms^1/2^/cm^2^ only enlarge the MMO regime mildly (Figure 7C). Large noise levels also cause the generation of MMO patterns in the regime where, without noise, the neuron exhibits STOs or fires tonically (σ = 0.1 µA×ms^1/2^/cm^2^; Figure 7D) or even when is silent (σ = 0.32 µA×ms^1/2^/cm^2^; not shown).

**Figure 8:**
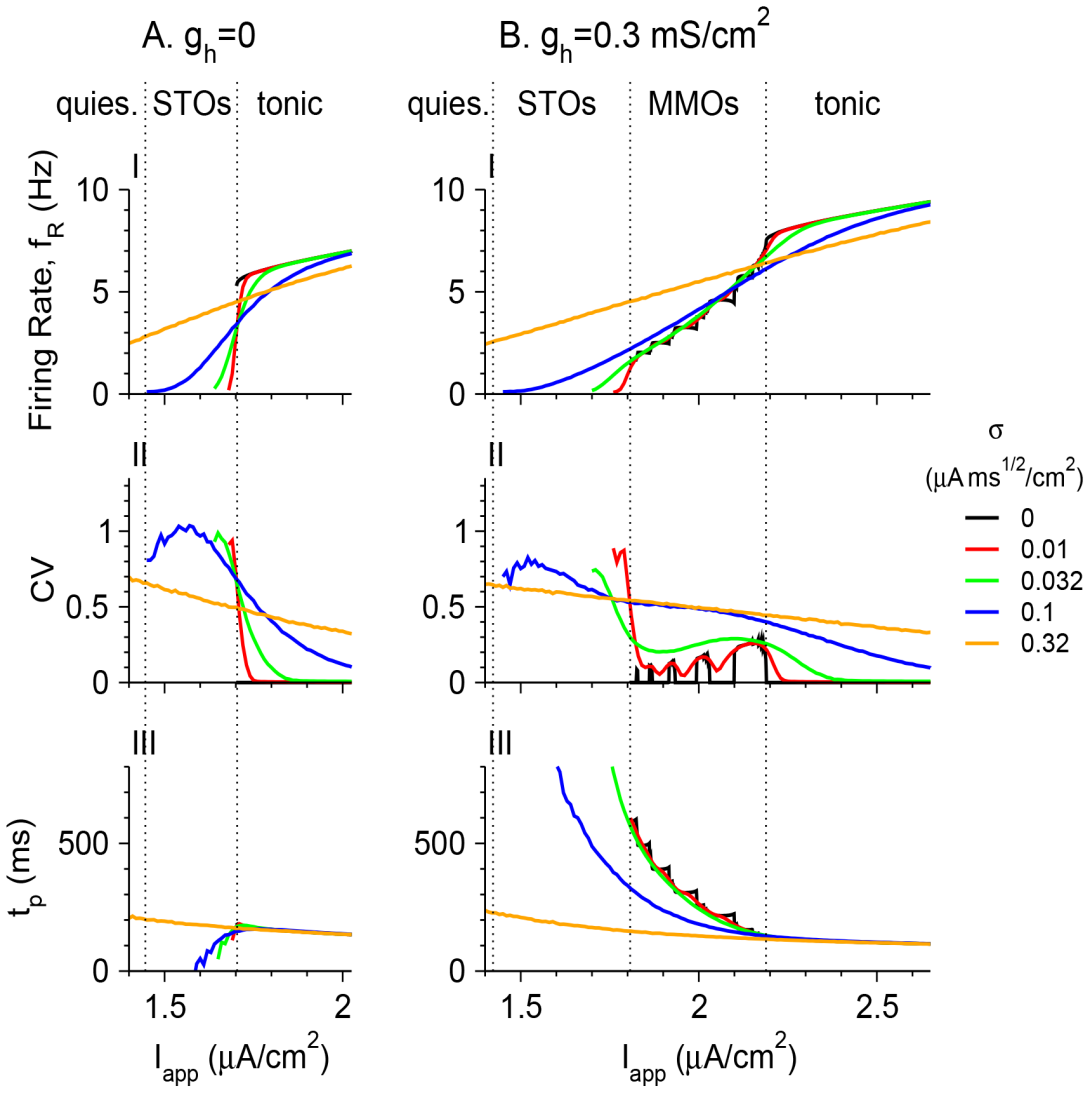
Properties of firing patterns without and with an intrinsic MMOs-generating mechanism. The firing rate *f*_R_ (I), the coefficient of variation CV (II) and the time period *t*_p_, computed assuming a Bernoulli process (Equation 3) (III) are plotted as a function of *I*_app_ for *g*_h_ = 0 (A) and *g*_h_ = 0.3 mS/cm^2^ (B). The colors of the lines denoting the values of σ (in µA×ms^1/2^/cm^2^) are: black – 0, red – 0.01, green – 0.032, blue – 0.1 and orange – 0.32. The vertical dotted lines denote the *I*_app_ values of the transitions between different dynamical states (quiescence, STOs, MMOs and tonic firing) of the noiseless neuron.

Effects of noise on the appearance of MMOs are also demonstrated by plotting the firing rate and the coefficient of variation (CV) of the model neuron as a function of *I*_app_ when there are no MMOs without noise (*g*_h_ = 0; Figure 8A_I,II_) and when the noiseless neuron can exhibit MMOs (*g*_h_ = 0.3 mS/cm^2^; Figure 8B_I,II_). In the first case, the regime of noise-induced MMOs is characterized by firing rates between that of tonic firing (around 6 Hz here) and 0, and by CV values between 0 and 1. For all levels of noise, the firing rate increases with *I*_app_ and CV decreases with it, since temporal maxima in *V*(*t*) correspond more frequently to spikes than to sub-threshold oscillations. A different situation is found when MMOs are observed without noise (*g*_h_ = 0.3 mS/cm^2^). For σ = 0, the firing rate as a function of *I*_app_ exhibits a Devil's staircase shape, and the CV fluctuates between 0 and positive values that have maxima around 0.08–0.28. This occurs because CV is 0 when the number of STOs between each pair of consecutive spikes is fixed; namely the state is 1^n^ for any n (with fixed n for all t; Figure 4C,E). As *I*_app_ increases, the neuron tends to fire in more complex periodic manner (Figure 4D), aperiodically (Figure 4F), or in modes such as n^1^ (figure 4G). In all such firing patterns, CV is positive. It goes back to 0 when the neuron switches to tonic firing (Figure 4H). Adding a small amount of noise smooths the firing rate versus *I*_app_ curve as well as the CV curve, and extends the *I*_app_ interval in which MMOs are observed on both sides. The CV is large (∼0.7–1) when MMOs begin to emerge as *I*_app_ increases, but then decreases as the firing patterns become similar to the MMOs observed for σ = 0. As *I*_app_ increases further, the CV increases as it follows a smooth version of the curve for σ = 0, and then decreases again to near zero as the firing pattern switches to tonic firing. The CV vs. *I*_app_ (or vs. *f*_R_) curve is therefore non-monotonous and has an N-shape form for moderate levels of noise. Only for large noise levels (σ = 0.1 µA×ms^1/2^/cm^2^ and above in Figure 8) does the CV decrease monotonically with *I*_app_ whereas the firing rate increases monotonically with it.

To further explore non-random firing patterns in the case where MMOs are generated by the effect of both an intrinsic mechanism and noise, the firing rate *f*_R_ and the coefficient of variation CV are computed in a simple model. In this simple model, the membrane potential *V* of a neuron oscillates between a high and a low voltage with a time period *t*_p_. When *V* reaches its maximal value, the neuron either fires a spike at random with a probability *p* or does not fire and goes back to its minimal value with a probability of 1-*p*. The spike duration is very small and refractoriness is neglected, such that the time period between two consecutive maxima of *V* does not depend on whether the peak occurs during a spike or a maximum *V* value of an STO, under a Bernoulli process [27]. The average inter-spike interval (<ISI> = 1/*f*_R_) is geometrically distributed (see “a simple model of MMOs generated by noise alone” in Methods) with an average *t*_p_/*p*, variance 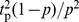 and CV 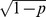. Therefore, for a Bernoulli process, 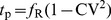
(3)Since the STO frequency that underlies MMOs depends only weakly on *I*_app_, if the spiking can in fact be well described by a Bernoulli process, then predicting *t*_p_ from *f*_R_ and CV should display a weak dependence on *I*_app_. In fact, for large σ (0.32 µA×ms^1/2^/cm^2^), *t*_p_ weakly decreases with *I*_app_, as takes place for tonic spiking with no noise (Figure 8A_III_ and 8B_III_, orange). In contrast, for small and moderate noise levels, *t*_p_ strongly increases as *I*_app_ decreases, by about a factor of 5, within the *I*_app_ regime where without noise, the model neurons exhibit MMOs (and even when they exhibit STOs). This behavior is caused by the low values of CV with respect to what is expected from a Bernoulli process, which stem from the fact that the MMOs are generated by an intrinsic neuronal mechanism. Note that for *g*_h_ = 0, the calculated values of *t*_p_ in the regime where the noiseless neurons exhibits STOs are below what is expected from Eq. 3. This is because CV is close to 1, beyond what is expected from a Bernoulli process. The model neuron tends to fire in clusters of spikes (Figure 7B_1_). An explanation of this behavior is beyond the scope of this article.

### Response of a motoneuron pool to constant and periodic inputs

**Figure 9:**
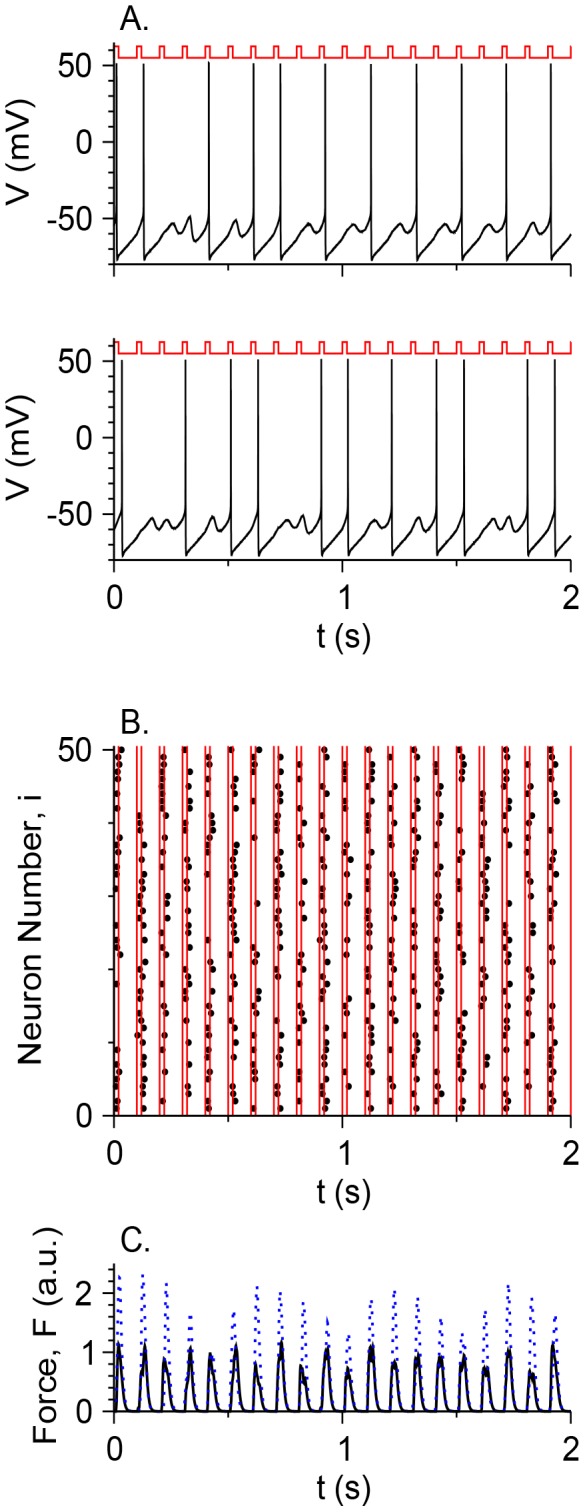
Response of a motoneuron pool to periodic stimulation from a CPG and neuromodulation. 50 uncoupled motoneurons are simulated, each receiving constant input *I*_app_ = 2 µA/cm^2^ mimicking the excitability effects of neuromodulators such as serotonin, and periodic stimulation from a CPG with a frequency *f* = 10 Hz. The periodic stimulation is *I*_c_ = 0.15 µA/cm^2^ during the first 20 ms of each cycle with a duration of *T*_per_ = 1/*f*, and 0 otherwise. Different neurons have different noise realizations. Additional parameters: *g*_h_ = 0.3 mS/cm^2^, σ = 0.032 µA×ms^1/2^/cm^2^. Realizations of the noise are different across motoneurons. (A) The membrane potential *V* as a function of *t* for two neurons (black). The stimulus pattern is schematically plotted above each panel (red) to emphasize the synchrony of spikes with the stimulus. (B) Rastergram of the spikes (black circles) of the 50 motoneurons. The stimulus is *I*_c_ between each pair of adjacent red lines. (C) The total force amplitude *F*, in arbitrary units (*A* = 1, Equation 4), generated by a whole muscle whose cells are innervated by the pool of motoneurons (black). The dotted blue line denotes a similar simulation with *I*_app_ = 2.4 µA/cm^2^.

There are about 50–100 motoneurons projecting to each vibrissa muscle [1], [4], with no chemical or electrical synapses among them. These neurons receive periodic stimulation from the vIRT or the Bötzinger nuclei [10], and are also slowly modulated by serotonin that increases their excitability [12]. To assess the ways in which the two stimuli control the activity of a motoneuron pool controlling cells in a single muscle, a simulation is tested on a pool of *N = *50 uncoupled motoneurons (*N* is the number of neurons) with *g*_h_ = 0.3 mS/cm^2^ and a noise level σ = 0.032 µA×ms^1/2^/cm^2^, that differ only by the realization of the noise (i.e., with different seeds for the noise of different neurons [28]). The excitability of the neurons, controlled naturally by neuromodulations and quantified here by *I*_app_, is within or around the MMOs regime. All the neurons are stimulated by the same periodic input, which is *I*_c_ during the first 20 ms of the period *T*_per_ = 1/*f* and 0 otherwise (Figure 9). For moderate values of *I*_c_ and values of *f* around the frequency of peaks (of STOs or spikes) of the non-stimulated neurons, the MMO nature of the dynamics is maintained, and the stimulus simply locks the spikes to its pace (Figure 9A). Since the firing frequency is lower than the peak frequency, different neurons with different noise realizations will not necessarily fire during the same stimulus period. If, on average, the neuron fires every *n* cycles of the stimulus, the number of spikes in each stimulus will be on average *N*/*n*, as shown in the rastergram in Figure 9B for *I*_app_ = 2 µA/cm^2^ and *I*_c_ = 0.15 µA/cm^2^. While most neurons fire during the “up” phase of the stimulus, some neurons fire somewhat later. To quantify the level of synchrony of the motoneuron pool, the total force developed in the muscle is computed. It is assumed that each spike, fired at time *t_s_*, generates a force twitch in the cells it innervates 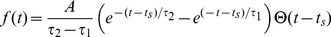
(4)where Θ is the Heaviside function, τ_1_ = 5 ms and τ_2_ = 6 ms [29] and *A* = 1 in arbitrary units. The force contributions of motor units (i.e., muscle cells that are innervated by the same motoneuron) are summed linearly. The total muscle force *F*, plotted in Figure 9C, shows that the motoneuron spikes are partially synchronized [30]. The amplitude, however, is about twice as small as the force amplitude developed for *I*_app_ = 2.4 µA/cm^2^, for which the isolated neuron fires every cycle (Figure 8C). These results reflect the following motor control scenario: when the non-stimulated motoneurons are in or around the MMOs regime, and motoneurons function under the effects of neuromodulators such as serotonin, the firing frequency is controlled by a moderate periodic frequency whereas the firing amplitude can be controlled by neuromodulators.

## Discussion

### Summary of the results

Experimentally, vibrissa facial motoneurons may fire in a mixed mode oscillation (MMO) state in response to constant current injection *I*_app_ [12], [14]. This firing mode affects the integration of synaptic and neuromodulatory inputs. This study investigated the generation of MMOs using a computational model [15]. A noiseless model does not exhibit MMOs without h conductances, but subthreshold oscillations (STOs) are obtained with moderate values of *g*_M_ and *g*_NaP_ (Figures 1,2). Addition of the h-type conductance *g*_h_ enables the generation of MMOs, with a firing frequency that depends on *I*_app_ like a Devi's staircase (Figures 3,4). The MMO range increases linearly with *g*_h_ (Figure 5). Small levels of stochastic noise increase the MMO regime by converting STOs or tonic firing to MMOs, often with CV values significantly lower than 1. Large noise levels generate MMOs with firing statistics indicating a Bernoulli process. Based on these results, it is possible to determine which one of three MMO mechanisms generates MMOs in a neuron based on intracellular recordings. This study shows how, by using the MMO mode, fast synaptic and slow neuromodulatory inputs can control the frequency and amplitude of whisking, respectively.

### Comparison of spinal and vibrissa motoneuron firing patterns

Mixed mode oscillations are found experimentally in both rat [18] and mouse [19] spinal motoneurons, as well as rat vibrissa motoneurons. In these cases, conductance-based models reveal MMOs as well. In experiments and models of the two types of motoneurons, persistent inward conductances such as *g*_NaP_ are needed to obtain MMOs [18]. In models of both types, adding noise extends the MMO range and makes the discharge more irregular. There are, however, major differences between the firing patterns of these two types of motoneurons. The firing rate of rat vibrissa motoneurons, as well as the MMO frequency, is about 5–10 Hz [7], [14], which is substantially lower than that of spinal motoneurons of rats [18] and mice [19], which is about 20–100 Hz. Therefore, the h-conductance with kinetics on the order of 100 ms, which is critical for generating MMOs in the model presented here, is not expected to play a major role in spinal motoneurons dynamics. In fact, it is sufficient to generate MMOs in a “minimal” model of spinal motoneurons with only transient Na^+^, delayed rectifier K^+^ and M-like K^+^ conductances [19], whereas in the present model additional g_NaP_ and *g*_h_ are needed. Another important difference has to do with the behavior just outside the *I*_app_ regime where MMOs emerge. In the present model the neuron exhibits STOs at *I*_app_ values just below those where MMOs exist. This behavior occurs because as *I*_app_ is increased, the rest state is first destabilized by a *supercritical* Hopf bifurcation (HB) (Figure 5), and MMOs are only observed after a period doubling (PD) bifurcation at a further larger *I*_app_ value. In contrast, in the spinal motoneuron model there is no STO regime, since the rest state is destabilized by a *subcritical* HB while a homoclinic trajectory arises (Shilnikov's homoclinic bifurcation scenario; see Figure 8 in [19]). Therefore, the existence of an STO regime points to which mechanism is responsible for intrinsic MMOs generation.

In the Shilnikov scenario, the dynamical system escapes spirally from an unstable fixed point [31]. This implies that for spinal motoneurons, the MMO amplitude increases from cycle to cycle until the neuron fires a spike, as shown both experimentally and in the model [19]. In contrast, in the present model the amplitudes of membrane potential oscillations remain constant between spikes (Figures 4,7). The method suggested here serves to determine whether MMOs are generated by the intrinsic mechanism based on *g*_h_ described in this work, or by that of [19]. Specifically, the intrinsic mechanism analyzed here exhibits a significant regime of STOs in *I*_app_ values below the MMOs regime, and the STOs amplitude is about constant with time. In the Shilnikov-based spinal motoneuron mechanism, there are no STOs for lower *I*_app_ values, and the STO amplitude increases with time. These different firing types can be distinguished based on intracellular recording.

### Distinguishing an intrinsic from a noise-induced mechanism

MMOs can be generated by an intrinsic mechanism, or by stochastic noise that transforms an STO state, a quiescent state, or a tonic firing state into an MMO state. The method presented here distinguishes between these two options based on recordings of the firing patterns for varying values of *I*_app_. The first step is to measure or compute the firing rate *f*_R_ and the coefficient of variation CV as functions of *I*_app_. An increase in *f*_R,_ together with a smooth decrease of CV as a function of *I*_app_ indicates that the STOs are generated by noise, although this cannot rule out the existence of an intrinsic mechanism for MMOs that is smeared out by large noise (Figure 8). If *f*_R_ increases with *I*_app_ and CV varies non-monotonically with *I*_app_, there is an intrinsic mechanism underlying the MMO firing pattern. Another method is to compute the time period *t*_p_ between peaks of oscillations (either STOs or spikes) assuming a Bernoulli process according to Equation 3. If there is a range of *I*_app_ where *f*_R_ rises by an order of magnitude while *t*_p_ decreases by the same order, this is an indication of an intrinsic mechanism.

### MMOs in various types of neurons and models

In the present model with *g*_h_ = 0, the transition between STOs and tonic firing is abrupt, reminiscent of a canard transition [32], [33] (Figure 1B). For *g*_h_>0, the STOs are destabilized via a PD bifurcation, MMOs are generated, and tonic firing is restored via an SNP bifurcation (Figure 3). A similar bifurcation scenario was observed in a model of fast-spiking cortical neurons [23]. That model did not include h-conductance, but rather implemented a K^+^ conductance *g*_Ks_ that was slow at hyperpolarized potential. MMOs were observed only if *g*_Ks_ was above a certain positive critical value. In contrast, in the present model, MMOs are observed in vibrissa motoneurons for any positive value of *g*_h_. The reason for this effect is unknown.

In addition to motoneurons, MMOs have been observed in other neuronal types. For example, MMOs with frequencies of a few Hz were observed in cortical interneurons [34], pyramidal cells of the frontal cortex [35], and stellate cells of the entorhinal cortex [36]–[38]. The conductances *g*_NaP_, *g*_M_ and *g*_h_ were found to be important for the generation of MMOs in the enthorhinal cortex [39], [40], and stochastic noise contributes to them as well [41], [42]. In another model of these neurons [43], STOs (but not MMOs) emerged from the interplay between *I*_NaP_ and *I*_h_. In the present model neither subthreshold oscillations nor mixed-mode oscillations are observed without *I*_M_. This difference between the present model and the one described in [43] stems from the fact that *θ*_r_, the half-maximum potential of the activation curve of *I*_h_ in the present model (see “Ionic currents of the model” in Methods) is about 14–17 mV more hyperpolarized than the resting potential, whereas *θ*_r_ in the Dickson et al. model [43] is more *depolarized* than at rest by 11–21 mV; hence the h current is more effective at membrane potentials near spike threshold. In the present model, the value for *θ*_r_ is consistent with the experimental observation by Hattox et al. [12] in vibrissa motoneurons, showing that the sag effect is substantial only much below resting potential. Mixed mode oscillations have been found in models of layer 5 pyramidal neurons [44] and in the Hodgkin-Huxley model of the squid giant axon when the activation time constants *τ*_h_ or *τ*_n_ were reduced, as a result of the “canard” effect [45].

### Functional significance

Control the whisking frequency and whisking amplitude in a coordinated manner, both between motoneurons that project to the same muscle and those that project to different muscles, is a challenge to the nervous system [46]. Since vibrissa facial motoneurons are uncoupled [8], and about 50–100 motoneurons project to each muscle, this coordination needs to emerge from the inputs to the motoneurons. The most obvious sources are the rhythmic input from the vIRt and Bötzinger nuclei in the brainstem [10], [11] and serotonergic modulation [12], although sensory feedback from the trigeminal ganglion [16] may affect motoneuron firing. If neurons are “standard” class I or II types [22], [47], neuromodulation can abruptly transform them from a silent state to a tonically active state, but the interaction of the rhythmic input with the tonically-firing neurons may generate complex firing patters. In their work on spinal motoneurons, Iglesias et al. [19] suggested that MMO patterns can be used to make the transition between quiescence and a high firing rate more moderate by reducing the neuronal gain. This effect is seen in the vibrissa motoneurons as well (Figure 8). In the whisker system, however, MMOs can serve another purpose.

Here, a control mechanism is suggested based on motoneurons that fire in an MMO mode (Figure 9). Moderate levels of periodic input control the population firing frequency. The serotonergic modulation controls the cell excitability and is modeled here by varying *I*_app_. Other possible effects of serotonergic modulation, which are not considered here, may have additional consequences for the network dynamics. The spiking activity of neurons is locked, although not fully, to the “up” state of stimulating periodic activity, whereas the number of STOs between spikes is controlled by the neuromodulation. Increasing the level of cell excitability will increase the number of neurons firing at each period, and, as a result, the whisking amplitude, but the population frequency will remain unchanged. If the intrinsic excitability of the motoneuron population is heterogeneous, the total number of neurons that fire can be controlled in a graded manner. The statistical nature of motor control is consistent with the fluctuations in whisking amplitudes from cycle to cycle [48]–[50]. In contrast, the fluctuations in the length of consecutive time periods, controlled by the periodic input, can be small, as demonstrated experimentally [4]. This control mechanism enables the division of labor among muscle cells, where each cell contracts once every few cycles [5], [51]. In this scenario, intrinsic cellular properties, phasic input and neuromodulation participate in controlling the frequency, phase and amplitude of whisking movement.

## Methods

### Ionic currents of the model

The following equations and parameters for the ionic currents are implemented [15]. Reference values of parameters are used unless otherwise stated.

Transient Na^+^ current, *I*_Na_: 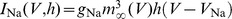, 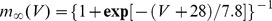, 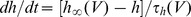, 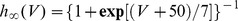, 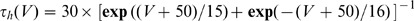, *g*_Na_ = 100 mS/cm^2^, *V*_Na_ = 55 mV.

Persistent Na^+^ current, *I*_NaP_; 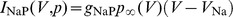, 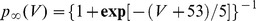, *g*_NaP_ = 0.04 mS/cm^2^.

Delayed rectifier K^+^ current, *I*_Kdr_: 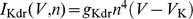, 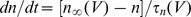, 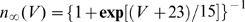, 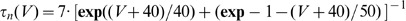, *g*_Kdr_ = 20 mS/cm^2^, *V*_K_ = −80 mV.

Slow AHP, Ca^2+^-dependent K^+^ current, *I*_AHP_: based on the work of [52], this activation current is modeled as a voltage-dependent activation with half-maximum potential above threshold. In this form, spikes are needed to activate the AHP channels. 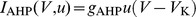, 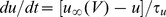, 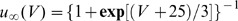, *g*_AHP_ = 10 mS/cm^2^, *τ*_u_ = 75 ms.

Slow voltage dependent, K^+^ current, *I*_M_: 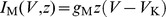, 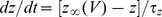, 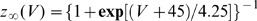, *g*_M_ = 1 mS/cm^2^.

Hyperpolarization-activated h-current *I*_h_: 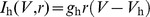, 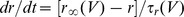, 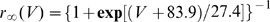, 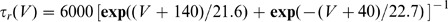, *V*_h_ = −27.4 mV *g*_h_ is usually set to 0 or 0.3 mS/cm^2^.

### Numerical methods

Simulations of differential equations without noise were performed using the fourth-order Runge-Kutta method with a time step of 0.01 ms implemented as a C program or within the software package XPPAUT [53]. Simulations of stochastic differential equations were conducted using the Euler method with the same time step. Simulations with a smaller time step (0.001 ms) did not reveal any observable differences. Bifurcation diagrams of deterministic dynamical systems were computed using XPPAUT.

### MMOs as a Bernoulli process

The analysis uses a neuron whose voltage oscillates at a time period *t*_p_. Each oscillation peak may become a spike with a probability *p* or a maximum of an STO with a probability of 1-*p*. For simplicity, the same *t*_p_ is assumed whether the first or second peak of the membrane potential is a spike or an STO. The inter-spike interval (ISI) equals *t*_p_ with a probability *p* and *nt*_p_ with a probability of 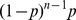 for an integer *n*≥2. For this Bernoulli process, the average ISI and ISI^2^ are therefore [27]

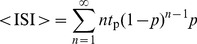
(5)




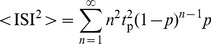
(6)where <…> represents time-average. I define *q* = 1-*p* and use the identities 
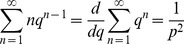
(7)




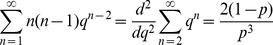
(8)to obtain 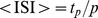, 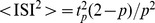, and therefore
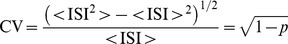
(9)

